# Thermal Conductivity in Mortar Samples with Copper Mine Tailings

**DOI:** 10.3390/ma18133157

**Published:** 2025-07-03

**Authors:** Lucas Daza, René Gómez, Ramón Díaz-Noriega, Roberto Gómez-Espina, Krzysztof Skrzypkowski, Oscar Jerez

**Affiliations:** 1 Faculty of Engineering, Universidad de Concepción, Concepción 4070415, Chile; 2 Faculty of Civil Engineering and Resource Management, AGH University of Krakow, 30-059 Krakow, Poland; 3 Institute of Applied Economic Geology, Universidad de Concepción, Concepción 4070415, Chile

**Keywords:** cement, mortar, copper mine tailings, thermal conductivity

## Abstract

The increasing generation of mine tailings poses significant environmental challenges, but their reuse in construction materials offers a sustainable solution by reducing the demand for natural aggregates. To advance the use of tailings in construction, the thermal conductivity of mortar samples incorporating copper mine tailings as partial replacements (10% to 50%) for coarse aggregates was investigated. The thermal conductivity was measured using the transient line source method, revealing a progressive increase from 0.32 W/m·K (control sample) to 0.52 W/m·K (50% replacement sample). The statistical analysis (ANOVA) confirmed significant differences among the sample groups, with the tailings content being a key factor. The higher thermal conductivity is attributed to the quartz-rich composition of the tailings, which enhances the heat transfer compared to conventional aggregates. The findings of this study contribute to demonstrating the feasibility of using mortar with copper tailings to modify the thermal conductivity of mortar.

## 1. Introduction and Literature Review

The growing demand for valuable metals and minerals from ore bodies has led to a significant global increase in mine tailings. To counter the increasing amount of mine waste, the incorporation of tailings into construction components has become a topic of great interest. Incorporating waste products into mortar is also attractive because it reduces the use of aggregates in mortar, which is important considering the environmental damage linked to sand extraction and cement production [1,2,3]. Then, given that the construction sector consumes 50% of the world’s natural resources, incorporating tailings into construction materials offers a viable solution to both manage mine wastes and reduce the consumption of natural, non-renewable resources by reintegrating these wastes into other production chains [4].

Historically, mine tailings have been managed by storing them in reservoirs, but these reservoirs have posed environmental and safety risks. For example, depending on their composition, tailings can generate acid mine drainage (AMD) [5]. Solutions to mitigate AMD, such as the use of fly ash, have proven effective due to fly ash’s neutralizing capacity and the formation of hydration products that serve as barriers against water and oxygen infiltration [6,7]. Approaches have also been suggested to enhance the efficiency of tailings disposal through innovative methods, such as densification, desulphurization, and co-disposal with waste rock [8]. More recently, however, practices have evolved to repurpose mine waste by altering the physical and chemical properties of tailings, including their application in cemented backfill [9,10,11], cement-based products [12,13,14,15], and brick production [16,17,18,19,20]. Tailings have also been used in geoengineering fields with the production of bentonite-tailings engineering barriers [21], tailings-based pavement subbases [22], and in compacted fill along with scrap tires [23]. Combining mine tailings as aggregates in mortar production offers another environmentally friendly option.

The literature on the thermal conductivity of mortar samples incorporating copper tailings—or other types of mining tailings—is relatively scarce. Most existing studies on mortars or concretes containing mine tailings primarily focus on mechanical strength (e.g., [24,25,26,27]). For instance, the incorporation of iron ore tailings into mortar has been evaluated, revealing a decrease in thermal conductivity (ranging from 0.41 to 0.76 W/m·K) attributed to changes in matrix morphology as the proportion of iron tailings increases [28]. A subsequent study applied these findings in a simulated building model to explore the practical implications [29].

Additionally, Chinese mine tailings, composed predominantly of SiO_2_ (71.8%), have been investigated as fine aggregates under varying water-to-binder (w/b) ratios [30]. The results indicate a reduction in thermal conductivity from 1.01 to 0.95 W/m·K as the w/b ratio increased from 0.3 to 0.5. In the case of copper mine tailings (used up to 20% and containing 47.7% Fe_2_O_3_ and 20.5% SiO_2_), it was observed that the degree of dewatering significantly influences thermal conductivity; while non-dewatered cement mortars showed increased thermal conductivity, dewatered samples exhibited reduced conductivity [31].

Fluorspar tailings (71.2% SiO_2_) have also been examined for use in cement mortars, particularly in geothermal backfill applications [32]. Furthermore, zircon mine tailings have demonstrated a potential thermal insulation capacity [33]. Electrodialytic technologies have been proposed for the removal of toxic elements from tailings-based mortars [34,35], enabling the incorporation of up to 50% mine waste without a clearly defined impact on thermal conductivity.

One consideration for the successful use of mine tailings in building or geothermal applications hinges on their ability to meet the requirements for thermal insulation materials. However, research remains limited, and further studies are needed to fully understand how different types of tailings (e.g., variations in mineralogy, particle size, and chemical composition) influence the thermal conductivity of mortar when incorporated as aggregates. To address this gap, an investigation was undertaken replacing 10% to 50% of coarse aggregates with copper mine tailings to evaluate the impact on thermal conductivity in mortar manufacturing.

## 2. Materials and Methods

The thermal conductivity of mortar samples was analyzed with different proportions of copper tailings replacing coarse aggregates. The thermal conductivity was measured using the transient line source. The objective was to explore an alternative use for copper tailings, assessing their feasibility as a replacement material in construction applications and its effect on thermal conductivity.

### 2.1. TLS-50 Equipment

**Figure 1:**
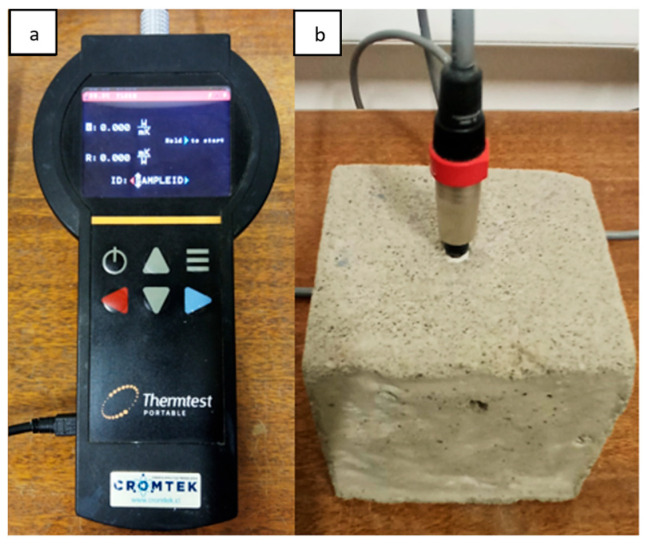
(**a**) TLS-50 equipment used to measure conductivity and thermal resistance on mortar samples. (**b**) Example of mortar sample with a previously drilled hole and a sensor needle with thermal paste inserted.

The needle method, also referred to as the transient line source (TLS) method, was used to measure the thermal conductivity of the samples. The durable design of the TLS probe allows both laboratory and field environments to be tested. Compared to other techniques, the TLS method can be applied to a wide variety of materials, including soils, rocks, concrete, polymers, moist and porous materials, and even liquids [36,37]. Furthermore, this method has proven to be highly effective in assessing porous materials that contain moisture [38]. The TLS-50 transient line source meter was employed as it produces an immediate and precise thermal conductivity assessment without damaging the samples (Figure 1). For this procedure, the 50 mm needle consisting of a heating wire and temperature sensor was fully inserted into each specimen for testing (Figure 1b). The main characteristics of the TLS-50 are described in Table 1.

**Figure 2:**
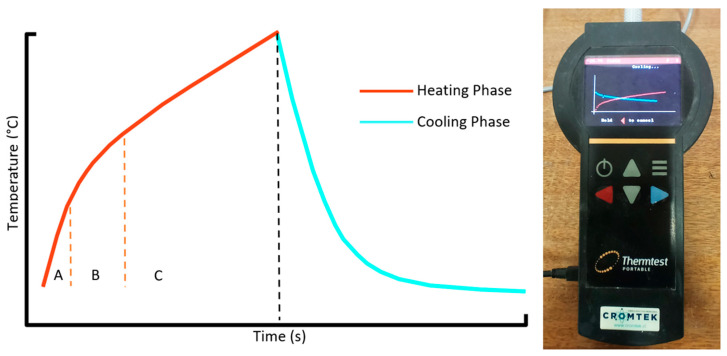
The typical form of the temperature-versus-time graph for the simple needle test. Heating and cooling phase equipment.

The thermal needle procedure used with the TLS-50 system involves two main phases (Figure 2—right). In the first phase, known as the heating stage, the needle is inserted into the sample and rests for a certain period, allowing it to reach a thermal equilibrium with its surroundings. Then, an electric current of known magnitude is applied, generating a constant heat flow. This process leads to an increase in the ambient temperature, which is recorded at short time intervals. The second phase, known as the cooling stage, begins when the heat source is turned off, and the subsequent decrease in temperature over time is recorded. A typical graphical representation of temperature versus time is shown in Figure 2—left. The heating and cooling stages can be further divided into three sub-stages [40]: (A) The initial seconds of the test, during which the system’s response is influenced by the thermal properties of the needle. (B) A semi-steady phase, during which the temperature variation follows a linear trend with respect to the logarithm of time. (C) The final stage, during which the temperature changes at the sample’s boundaries begin to deviate from one of the fundamental assumptions of the method.

The TLS-50 equipment delivers heat to the sample by a constant current source (*q*), and the temperature increment is recorded over a defined period. The thermal conductivity (*k*) is calculated using the slope (*a*) of the curve between the temperature increment and the logarithm of time using Equation (1). The higher the thermal conductivity of a sample, the less steep the slope will be [39].

(1) $$k=\frac{q}{4\pi a}$$

For samples of hard material, such as rock and concrete, a 50 mm test needle and a 4 mm diameter bit must be used. During the tests, heat-dissipating grease is used to optimize the contact between the sensor and the sample.

### 2.2. Materials

**Figure 3:**
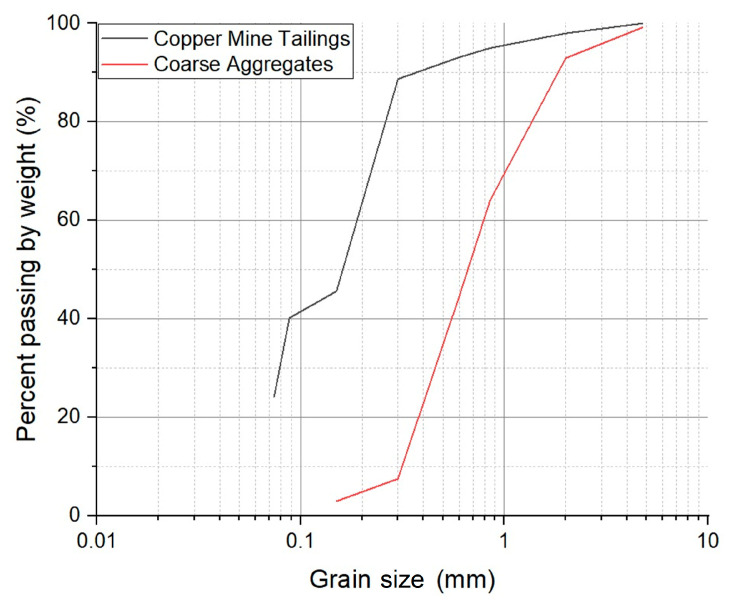
Particle size distribution of coarse aggregate and copper mine tailings.

Cubic mortar samples with tailings were used in this study. The mortar samples were fabricated using cement, coarse aggregate, water, and copper mine tailings. Table 2 and Figure 3 show the particle size distribution of the mine tailings and coarse aggregate used. Here, the particle size distribution of the mine tailings used is finer than that of the aggregate.

#### 2.2.1. Cement

**Figure 4:**
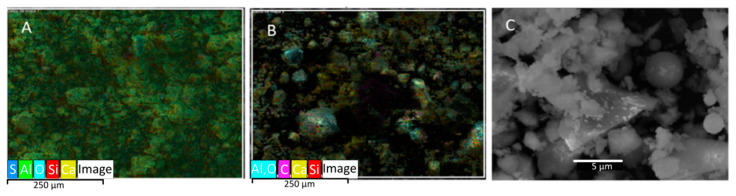
SEM of pozzolanic cement with WD = 10.4 mm ((**A**,**B**) scale of 250 μm (Mapping); (**C**) scale of 5 μm).

A pozzolanic-class cement was used based on its composition and strength. Since the thermal conductivity of mortar is relatively low, it acts as an insulator. Commonly, in a large mortar mass, hydration can lead to a significant temperature rise [41], causing exothermic hydration reactions. Figure 4 shows Scanning Electron Microscopy (SEM) morphologies of the cement used at scales of 250 and 5 µm, demonstrating the wide range of sizes of the particles with irregular morphologies and the small spherical pozzolana particles.

**Figure 5:**
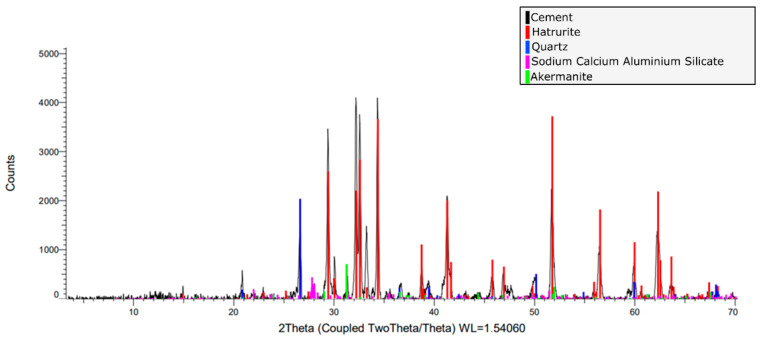
X-ray diffraction (XRD) of pozzolanic cement. The overlap of the diffraction peaks of the main components in the range of 2θ = 3° to 70° are 2 Theta (Coupled TwoTheta/Theta) WL = 1.54060.

The mineral phases present in the cement used were determined from an X-ray diffraction analysis (Figure 5). In order of abundance, Hatrurite (68.7%), quartz (14.5%), Plagioclase (10.2%) and Akermanite (6.6%) appear. Both Hatrurite (Ca_3_SiO_5_) and Akermanite (Ca_2_MgSi_2_O_7_) confer cementitious properties to the material. This mineralogy is consistent with the abundant calcium observed through the EDS analysis.

#### 2.2.2. Aggregates

**Figure 6:**
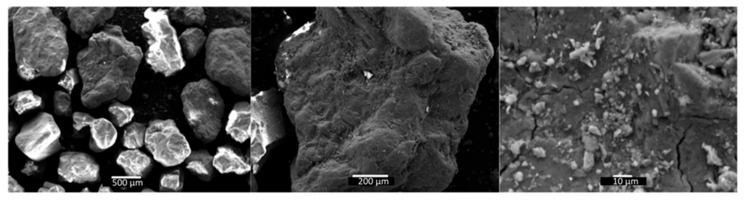
SEM of coarse aggregate—scales of 500 μm, 200 μm, and 10 μm.

The maximum particle size used in this study was less than 4.5 mm for the coarse aggregate. The coarse aggregates had a density of 2.77 g/cm^3^ [42]. When sand is completely dry, thermal conduction is dominated by the contact points between solid particles. Figure 6 shows the SEM morphologies of the coarse aggregate at scales between 500 µm and 10 µm. The irregular morphology of the surface of the grains and their angularity can be observed, as well as the gradation in their particle size. Based on the roundness scale of Powers [43], the particles are subrounded and have low sphericity.

**Figure 7:**
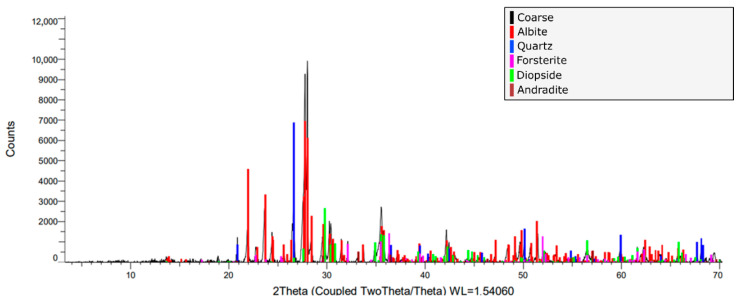
X-ray diffraction pattern of coarse aggregate (red—albite, Blue—quartz, Green—diopside, Pink—forsterite, and Maroon—andradite). The overlap of the diffraction peaks of the main components in the range of 2θ = 3° to 70° are 2 Theta (Coupled Two Theta/Theta) WL = 1.54060.

Five types of mineral components were obtained by X-ray diffraction (Figure 7): albite (64.6%), diopside (13.1%), quartz (11.8%), forsterite (8.5%), and andradite (1.7%). The overlap of the diffraction peaks of the main components in the range of 2θ = 3° to 70° can also be observed in Figure 7. The thermal conductivity of albite, the most abundant phase, ranges from 2 to 2.3 W/m·K at room temperature, whereas that of quartz reaches higher values, between 6.2 and 11.2 W/m·K [44]. The morphological characteristics, together with the aforementioned mineralogical composition, suggest a mafic volcanic origin of the source rock of the aggregates, which have been fragmented, transported, and accumulated naturally by water currents.

#### 2.2.3. Copper Mine Tailings

**Figure 8:**
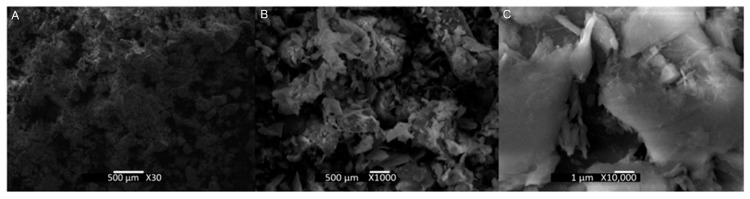
SEM with EDS of copper tailings ((**A**), (**B**), (**C**): scales of 500 μm, 10 μm, and 1 μm).

Figure 8 presents the morphologies of the copper mine tailings. The morphology of the particles is irregular due to the comminution processes. The particle sizes were mostly less than 500 µm, with finer sizes predominating, in accordance with the particle size distribution presented in Figure 3. The tailings used in this study had a bulk density of 2.67 g/cm ^3^, determined by pycnometry [42].

In Table 2, the granulometric characteristics of the copper tailings are presented, indicating a relatively fine material. The particle size distribution can be described using the characteristic diameters D_80_, D_60_, D_30_, and D_10_, representing the particle diameters below which 80%, 60%, 30%, and 10% of the material, respectively, pass through. The coefficient of uniformity (cu) is 3.39, suggesting a narrow particle size range. The coefficient of curvature (cc) is 0.59, which, along with the cu value, provides insight into the gradation and suitability of the material. The chemical composition of the mine tailings was obtained by X-ray fluorescence (XRF) and is shown in Table 3.

**Figure 9:**
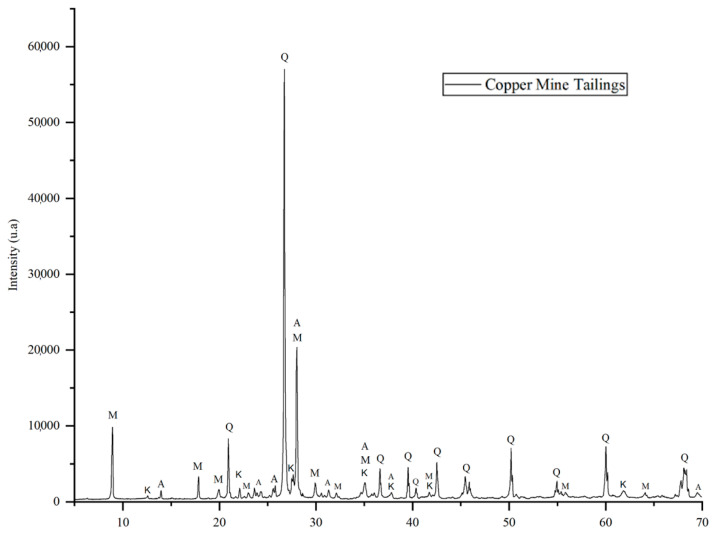
The mineralogy of copper mine tailings (DRX). The overlap of the diffraction peaks of the main components in the range of 2θ = 3° to 70° are 2 Theta (Coupled Two Theta/Theta) WL = 1.54060. Q: Quartz, M: Muscovite, A: Albite, K: Kaolinite.

Figure 9 shows the corresponding diffractogram of the copper tailings, depicting their respective phases. The mineral phases present are quartz (Q, 43.9%), albite (A, 23.5%), muscovite (M, 21.1%), and kaolinite (K, 11.5%). This composition indicates a felsic-type parent rock, with a certain degree of alteration that gives rise to the appearance of kaolinite, unlike the aggregates used, which originate from mafic rocks. Muscovite has a thermal conductivity of 2.3 to 3.9 W/m·K, similar to that of albite [44]. The presence of abundant quartz is important because of its potential influence on thermal conductivity and because it is a resistant mineral, even in the alkaline conditions of the medium presented by the mortars [45]. Elevated quartz and silica (SiO_2_) concentrations typically result in higher thermal conductivity, whereas aluminosilicate phases exhibit an inverse relationship with conductive heat transfer [46].

## 3. Sample Preparation and Testing Procedures

Six mortar cubes were prepared with aggregate replacements using copper mine tailings in ranges from 10% to 50%, with a water–cement ratio of 0.57. This percentage was chosen because it has shown good results in the mechanical performance of mortar [47]. In addition, a control sample (control) without tailings was fabricated. The cubic samples had a length of 105 mm (Figure 1b). The curing process was carried out for 28 days in water at a temperature of 20 ± 1 °C. The cubic samples were subsequently dried in an oven for 24 h at 110 ± 5 °C and then allowed to cool to 21 ± 2 °C. This allowed the samples to be tested under the same humidity conditions; however, it is important to note that this drying can favor the generation of micro-cracks and changes in the strength of the mortar. Details of the mortar samples are presented in Table 4.

Once the mortar cubes were prepared, a 4 mm diameter nail was inserted to create a hole in the center of the samples. The test needle was coated with thermal paste to enhance sensitivity and ensure a better thermal contact between the wave sensor and the dry mortar sample before using the TLS-50.

The particle size distribution analysis of the samples (Table 5) revealed important trends in the curvature (cc) and uniformity (cu) coefficients with different percentages of copper tailings replacement. The uniformity coefficient (cu), ranging from 2.0 to 8.11, showed a consistent increase as the replacement blends were increased from 10% (10 RCR) to 50% (50 RCR), indicating a progressively broader particle size distribution with the higher tailings content.

**Figure 10:**
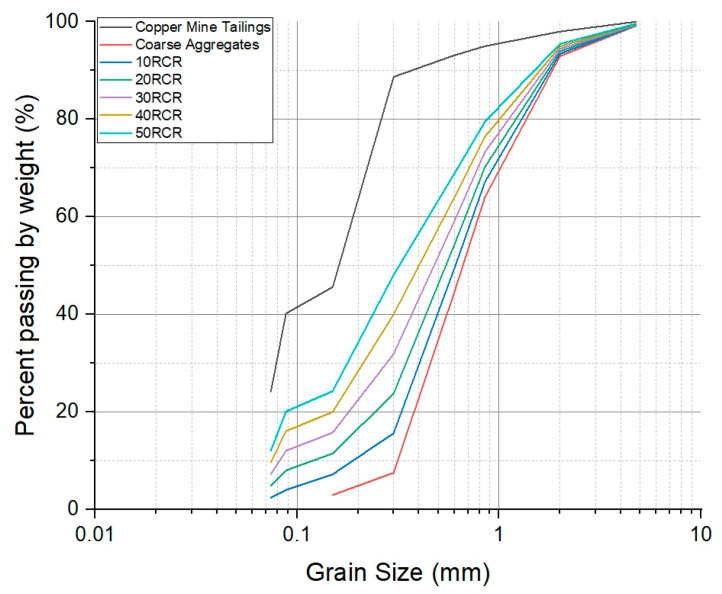
Particle size distribution of coarse aggregate and copper mine tailings with different replacements.

Figure 10 shows the particle size distribution curves for the coarse aggregate, copper mine tailings, and replacement blends from 10% to 50% (10 RCR–50 RCR). The coarse aggregate displays a distribution dominated by particles larger than 1 mm, while the mine tailings show a significant fine particle content. As the replacement percentage increases, the curves progressively shift towards the fine particle region, demonstrating a gradual transition from a coarse gradation to one more closely resembling pure tailings.

Table 6 shows the percentages of the mineralogical proportions in the samples. The coarse aggregates are predominantly composed of albite (64.6%), with minor contributions from diopside (13.1%), quartz (11.8%), and forsterite (8.5%). In contrast, the copper mine tailings exhibit a high content of quartz (43.9%) and phyllosilicates, such as muscovite (21.1%) and kaolinite (11.5%). As the replacement percentage increases, a progressive reduction in albite (60.5% to 44.1%) is observed, along with an increase in quartz (15% to 27.9%), muscovite (2.1% to 10.6%), and kaolinite (1.2% to 5.8%). These mineralogical proportions need to be considered when analyzing the thermal conductivity values of the different sample mixtures.

## 4. Results and Discussions

### Mortar Thermal Conductivity

**Figure 11:**
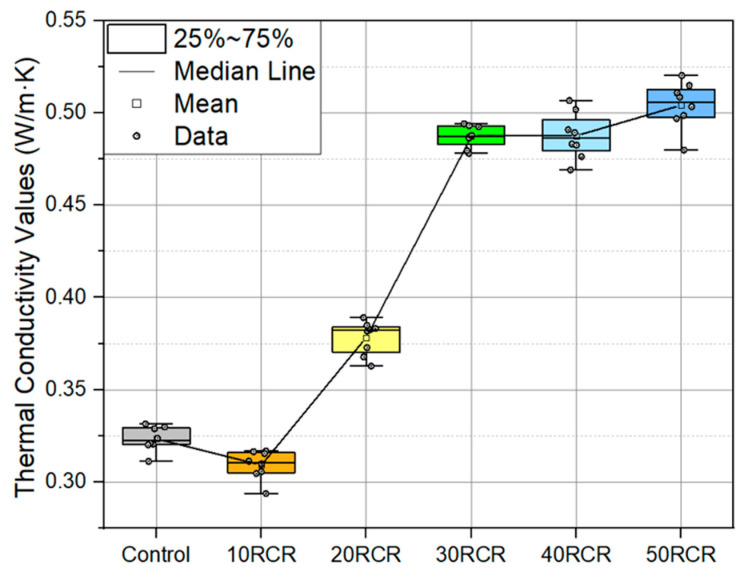
The thermal conductivity of the mortar samples, with eight measurements per sample.

The thermal conductivity results are shown in Figure 11 using a box plot for the samples. An increasing trend in thermal conductivity can be observed as the percentage of tailings rises, progressing from approximately 0.32 W/m·K in the control sample to 0.52 W/m·K in the 50% replacement sample. The dispersion of and the variation in the data within each group are relatively low. These results indicate that the use of copper tailings increases the thermal transfer capacity of mortar, which should be considered depending on the intended application of the mortar. This correlation can be related to the quartz content increment in the samples, as quartz exhibits the highest thermal conductivity among the aggregate minerals.

Table 7 presents the descriptive statistics for the six sample groups, with eight measurements for each using the TLS-50 equipment. The mean thermal conductivity values ranged from 0.309 (10 RCR) to 0.504 (50 RCR), indicating systematic differences between the groups. The standard deviations were low (0.006–0.013), showing consistent measurements within each group. The coefficients of variation were moderate (1.23–2.56%), implying a homogeneous variance across the groups. Most of the sample groups show a negative skew, indicating left-skewed distributions.

The D_50_ of the copper tailings used in this study shows a similar value to that reported in previous studies [48,49,50]. Therefore, these values cannot be considered to be particularly high or low in relative terms, except in the case of [51], in which the D_50_ value was significantly lower (0.031 mm). In other studies, such as Fjellerup et al. [52] and Zhou et al. [53], a larger particle diameter tended to be associated with higher thermal conductivity. However, based on the available values in this study, it is not possible to accurately predict the thermal behavior of the material, since other factors—such as the chemical and mineralogical composition of the sample—must also be considered for a comprehensive analysis.

In Table 4, it can be seen that the densities between the samples are quite similar. Even materials with identical densities can exhibit varying thermal conductivity values when different aggregates are used because the mineral composition of each aggregate type has distinct thermal properties. The mineralogical phases of the aggregates largely determine the thermal conductivity for normal-weight cementitious materials. However, with lightweight cementitious materials, the number of air voids and the moisture content mask the effect of the aggregate type [54]. The samples in this study exhibited thermal conductivity values similar to that of lightweight concrete. Furthermore, it should be noted that the aggregate content in mortar can vary significantly, ranging from two to nine times the amount of cement, which influences its mechanical properties in general and may impact its thermal properties as well. In particular, the tailings used have been shown to exert a slight influence on the compressive strength of mortar, as was also reported in a previous study [47].

**Figure 12:**
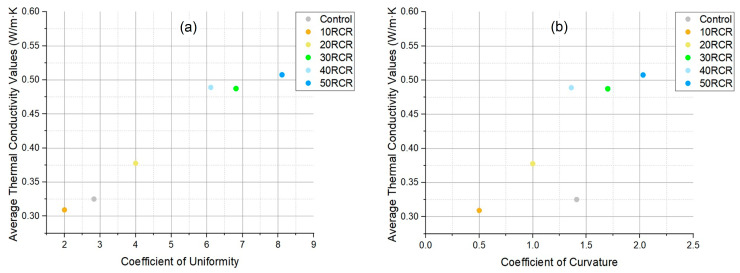
(**a**) Thermal conductivity based on the coefficient of uniformity, and (**b**) thermal conductivity based on the coefficient of curvature.

As noted, the type of aggregate can significantly influence the thermal conductivity of concrete due to differences in structural and mineralogical properties. Generally, sand has a greater thermal conductivity than cement paste, and consequently, the thermal conductivity of cement concrete in a dry state increases with the increasing volume fraction of sand [55]. Additionally, a higher number of interfacial zones, resulting from the finer particle size of the aggregate, contributes to lower thermal conductivity, an effect primarily attributed to the reduced contact area between the sand and the cement matrix [28]. This phenomenon explains why the 10 RCR sample (with 10% replacement) exhibited lower thermal conductivity than the control, as the reduced sand quantity dominates at this stage. However, beyond 20% replacement, this effect is reduced as the chemical and mineralogical composition of the copper tailings (particularly their high quartz content, as shown in Figure 9) becomes dominant, leading to increased thermal conductivity compared to the control. Furthermore, improved particle arrangement would enhance the interparticle contact and conductive heat transfer, correlating with elevated cc and cu values. The relationship between the average thermal conductivity of the samples and the uniformity and curvature coefficients can be seen in Figure 12. Notably, a direct and nearly linear correlation is evident, especially with respect to the coefficient of uniformity.

Figure 3 shows that the copper mine tailings consist of smaller grains (Figure 3) compared to the coarse aggregates in this study. Studies like [56] have shown that thermal resistivity decreases as particle size increases because coarser materials facilitate better grain-to-grain contact and reduce void spaces. Well-graded, rounded sand can be more densely packed, resulting in a higher thermal conductivity than that of angular or poorly graded tailings. This aligns with observations that granular materials with more contact points per particle exhibit improved heat transfer.

**Figure 13:**
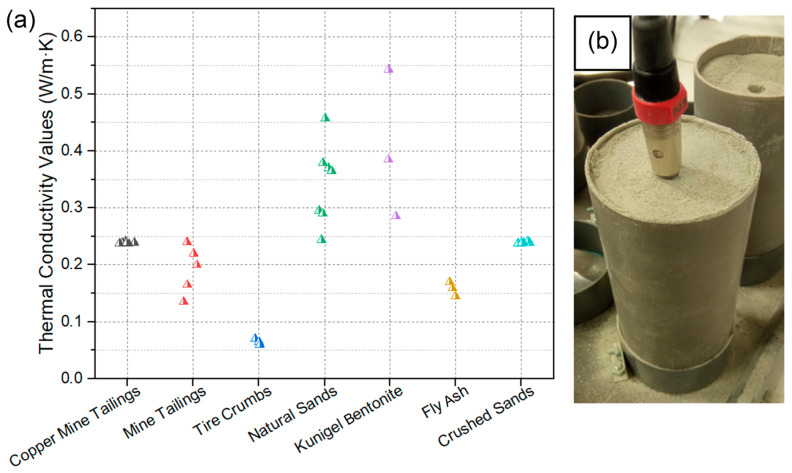
(**a**) Thermal conductivity values from different studies (Modified from [5])—copper mine tailings (this study), mine tailings and tire crumbs [23], natural sands [58], Kunigel bentonite [56], fly ash [59], and crushed sands [60]. (**b**) Compacted copper tailings being measured with the thermal needle.

In addition, Figure 13 shows the thermal conductivity values of the copper mine tailings used in this study and similar studies using other materials. Here, the thermal conductivity of the mortar with copper tailings was lower than that reported for natural sand when similar transient, needle-based techniques were used with both [57]. This discrepancy can be attributed to differences in their moisture content, particle size distribution, and density. In this study, the copper mine tailings were tested in a dry state, whereas the natural sands were typically evaluated at or near their optimum moisture content. Since water (165 °C·cm/W) has a significantly lower thermal resistivity than air (4000 °C·cm/W), the replacement of air with water in soil pores enhances their thermal conductivity [58]. This explains why the dry tailings exhibit lower conductivity than the moist natural sands. Thermal resistivity sharply increases with slight reductions in the moisture content, reinforcing the importance of testing conditions when comparing materials [57]. Variations in testing methods, like the needle probe used in this study and the thermistor simulations used in [56], may introduce minor discrepancies. However, the trends remain consistent: dry, fine-grained materials, like tailings, exhibit lower conductivity than moist, coarse-grained ones, like sands.

## 5. Analysis of Results

A one-way ANOVA model was used to analyze the results, comparing three or more levels within a single factor [61]. This model requires the following statistical assumptions: (1) The independence of observations: samples must be mutually independent, with no underlying correlations. (2) Normality: the data values in each group should follow a normal distribution. (3) The homogeneity of variances (homoscedasticity): variability across groups should be similar.

The ANOVA test aimed to verify the null hypothesis that the means of all levels are equal, i.e., increasing the percentage of tailings in the mortar does not affect its thermal conductivity values. The analyses were performed using the software Origin Lab Version 9.0. The F-statistic, with degrees of freedom k − 1 and n − k, where k is the number of groups (*k* = 6), and n is the number of observations (*n* = 8), was applied. Additionally, the *p*-value was used to determine whether the result was statistically significant, typically using a threshold of 0.05.

Since the *p*-value of the F-test is less than 0.05, there is a statistically significant difference among the means of the six variables at the 5% significance level (Table 8), meaning that the copper mining tailings replacement does affect thermal conductivity.

### 5.1. Normality of the Data

**Figure 14:**
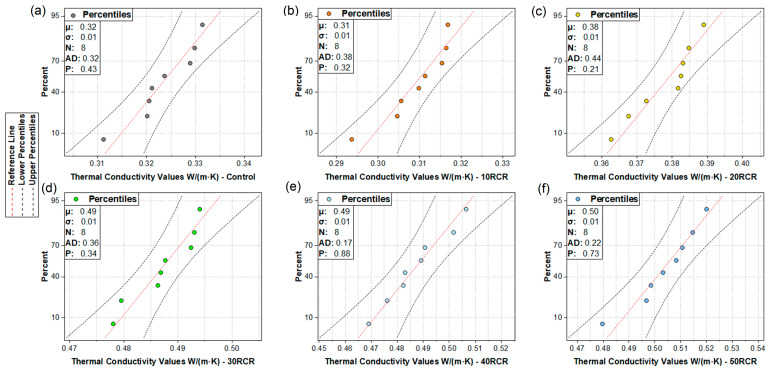
Normal probability plots of sample results: (**a**) control sample, (**b**) 10% tailings replacement, (**c**) 20% tailings replacement, (**d**) 30% tailings replacement, (**e**) 40% tailings replacement, and (**f**) 50% tailings replacement.

To assess the normality of the data, the Anderson–Darling test was used. This is a goodness-of-fit test that evaluates how far the data deviates from a theoretically expected normal distribution. In Figure 14, normal probability plots are shown. Each plot includes the mean (μ), standard deviation (σ), number of measurements, and the AD (Anderson–Darling) value. The closer the AD value is to zero, the closer the data distribution is to a normal distribution. Across all the plots, the highest AD value is 0.439, which, although it indicates some deviation, is not considered critical and does not involve any outliers. Furthermore, each plot reports a *p*-value of less than 0.05, supporting the assumption that the data do not significantly deviate from normality.

The Shapiro–Wilk test, which is one of the most used tests for assessing normality, was also performed (Table 9). This test is based on comparing the sample quantiles to those of a fitted normal distribution.

Since the *p*-values obtained from the normality tests are greater than or equal to 0.05, there is no evidence to reject the null hypothesis that the samples come from a normal distribution. Therefore, at a 95% confidence level, the data can be considered to follow a normal distribution, confirming the assumption of normality.

### 5.2. Homogeneity of Variance

In a variance analysis, the assumption of homoscedasticity must be satisfied. The homogeneity of variances assumption was validated using the Levene′s test (Table 10).

This test supports the results obtained from the skewness and kurtosis values. In this case, the *p*-value is 0.2498 (greater than 0.05), indicating that there is no statistically significant difference between the variances. Since the *p*-value is greater than 0.05, it can be concluded that the variances are not significantly different, meaning the variances of the compared groups are statistically equal. So, when comparing the variances of groups such as the control—50 RCR—the absence of significant differences suggests that the spread of the data around the mean is similar across all the groups. Therefore, the assumptions are satisfied, which indicates that increasing the replacement of the samples increases their thermal conductivity.

### 5.3. Multiple Range Test

To complement the analysis, Fisher’s Least Significant Difference (LSD) procedure was used to distinguish between the means. With this method, there is a 5.0% risk of concluding that each pair of means is significantly different when, in fact, the true difference is zero.

**Figure 15:**
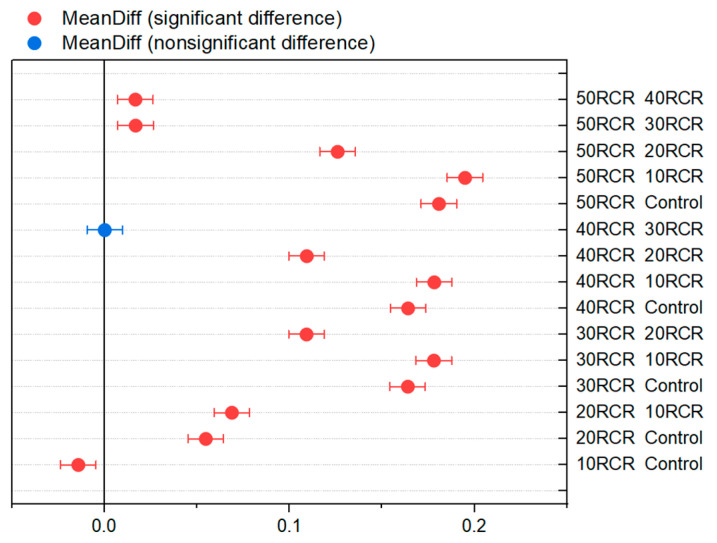
Means comparison—Fisher LSD.

Of the 15 pairs, 14 show statistically significant differences at a 95.0% confidence level, indicating that almost all the samples belong to different groups. The only exception is the pair 30 RCR and 40 RCR, which belong to the same homogeneous group (Figure 15). This result demonstrates that increasing the percentage of copper mine tailings in the samples increases the thermal conductivity, except that between 30 RCR and 40 RCR, there is almost no change in the thermal conductivity performance. In other words, the null hypothesis that all the means are equal is rejected, and the alternative hypothesis that one or more of the means are different is accepted.

## 6. Conclusions

In this experimental study, the thermal conductivity of mortar with copper mine tailings (10 to 50% replacement of coarse aggregates) was evaluated, using the transient line source method, complemented by material characterization through EDS, SEM, and XRD analyses. The main conclusions are the following:

- The TLS methodology proved effective for precise thermal measurements in mortar samples with tailings.
- Mineralogical characterization confirmed the quartz-rich composition (43.9%) of tailings as the key factor influencing their thermal behavior.
- The results demonstrated a significant increase in thermal conductivity (0.32 to 0.52 W/m·K) with a higher tailings content, establishing clear differences between the replacement levels, except for the range between 30 and 40%, in which no change was observed. Statistical validation confirmed these trends were significant, with *p* < 0.05.

This work provides both a methodological framework and practical data for utilizing copper tailings in construction materials, offering a dual solution for waste management and improved thermal performance in mortar applications. The findings could serve as a valuable reference for future research on tailings valorization and sustainable material development in the mining and construction sectors.

## Figures and Tables

**Table 1:** Parameters and description of TLS-50 equipment [39].

Materials	Mortar, Rocks, and Polymers
Measuring Capacity	Bulk Properties
Thermal Conductivity	0.3 to 5 W/m·K
Thermal Resistivity	0.2 to 3.3 m·K/W
Measuring Time	1 to 90 min
Precision	±2%
Temperature Range	−40 to 100 °C

**Table 2:** Particle size parameters of the coarse aggregates and mine tailings.

Parameter	Coarse Aggregate	Copper Mine Tailings
D_80_ (mm)	1.36	0.26
D_60_ (mm)	0.79	0.19
D_30_ (mm)	0.48	0.079
D_10_ (mm)	0.31	0.056
cc	0.84	0.59
cu	2.55	3.39

**Table 3:** Reported chemical composition of copper mine tailings (%).

Type of Tailings	Source	SiO_2_	Al_2_O_3_	Fe_2_O_3_	CaO	MgO	SO_3_	K_2_O	Na_2_O
Copper tailings	Porphyry (Atacama, Chile)	66.71	20.47	1.84	0.36	1.39	0.52	6.15	1.54
		**P_2_O_5_**	**TiO_2_**	**Cr_2_O_3_**	**MnO**	**CuO**	**Rb_2_O**	**ZrO_2_**	**BaO**
		0.16	0.52	0.04	0.02	0.10	0.02	0.02	0.07

**Table 4:** Main parameters and characteristics of mortar cubes samples.

Sample Name	Aggregate Replacement (%)	Materials (%)				28-Day Density (kg/m^3^)	
		Cement	Coarse Aggregate	Mine Tailings	Water	Wet	Dry
Control	0	27.27	57.23	0	15.5	2761	2165
10 RCR	10	27.27	51.51	5.72	15.5	2783	2250
20 RCR	20	27.27	45.78	11.45	15.5	2667	2120
30 RCR	30	27.27	40.06	17.17	15.5	2713	2102
40 RCR	40	27.27	34.34	22.89	15.5	2809	2217
50 RCR	50	27.27	28.62	28.62	15.5	2831	2190

**Table 5:** The values of the coefficients of curvature (cc) and uniformity (cu), respectively, for evaluating the particle size distribution and the classification of granular materials, considering the coarse aggregates and mine tailings.

Sample	cc	cu
10 RCR	0.50	2.0
20 RCR	1.0	4.0
30 RCR	1.7	6.82
40 RCR	1.36	6.11
50 RCR	2.03	8.11

**Table 6:** The percentages of the mineralogical proportions in the coarse aggregate, copper mine tailings, and different replacements.

Mineral (%)	Coarse Aggregate	Mine Tailings	10 RCR	20 RCR	30 RCR	40 RCR	50 RCR
albite	64.6	23.5	60.5	56.4	52.3	48.2	44.1
diopside	13.1	-	11.8	10.5	9.2	7.9	6.6
quartz	11.8	43.9	15	18.2	21.4	24.6	27.9
forsterite	8.5	-	7.7	6.8	6	5.1	4.3
andradite	1.7	-	1.5	1.4	1.2	1	0.9
muscovite	-	21.1	2.1	4.2	6.3	8.4	10.6
kaolinite	-	11.5	1.2	2.3	3.5	4.6	5.8
Total	100	100	100	100	100	100	100

**Table 7:** Descriptive statistics of the thermal conductivity for the different samples.

Sample	Count	Mean	Standard Deviation	Coefficient of Variation	Standardized Skewness	Standardized Kurtosis
Control	8	0.323	0.0067	2.06	−0.6703	0.113
10 RCR	8	0.309	0.0078	2.53	−1.301	0.680
20 RCR	8	0.378	0.0092	2.44	−0.826	−0.502
30 RCR	8	0.487	0.0060	1.23	−0.596	−0.588
40 RCR	8	0.489	0.013	2.56	0.264	−0.333
50 RCR	8	0.504	0.013	2.50	−1.003	0.590

**Table 8:** Summary of ANOVA results and model error.

	Sum of Squares	Degrees of Freedom	Mean Square	F Value	*p* Value
Model	0.315	5	0.0629	697.86	0.0001
Error	42	0.0038	9.02 × 10^−5^		

**Table 9:** Shapiro–Wilk test results.

Sample	Statistic	*p*-Value	Decision at Level (5%)
Control	0.9255	0.47602	Cannot reject normality
10 RCR	0.89068	0.23748	Cannot reject normality
20 RCR	0.90219	0.3023	Cannot reject normality
30 RCR	0.90141	0.29748	Cannot reject normality
40 RCR	0.97125	0.90761	Cannot reject normality
50 RCR	0.95308	0.7422	Cannot reject normality

**Table 10:** Homogeneity of variance test.

Levene′s Test (Absolute Deviations)	DF	Sum of Squares	Mean Square	F Value	Prob > F
Model	5	1.95162 × 10^−4^	3.90325 × 10^−5^	1.38361	0.24991

## Data Availability

The original contributions presented in this study are included in the article. Further inquiries can be directed to the corresponding authors.
